# Supplementary material for: Origin of aromatase inhibitory activity via proteochemometric modeling
Source: PeerJ. 2016 May 12;4:e1979. doi: 10.7717/peerj.1979 (PMC4868594; doi:10.7717/peerj.1979)
Supplement: Data S1 [file peerj-04-1979-s001.pdf]

Supplementary Information

# Unraveling the origin of aromatase inhibitory activity via proteochemometric modeling

Saw Simeon[1], Ola Spjuth[3], Maris Lapins[3], Sunanta Nabu[1], Nuttapat Anuwongcharoen[1,3], Virapong Prachayasittikul[2], Jarl E. S. Wikberg[3], and Chanin Nantasenamat*[1]

[1] *Center of Data Mining and Biomedical Informatics, Faculty of Medical Technology, Mahidol University, Bangkok 10700, Thailand*

[2] *Department of Clinical Microbiology and Applied Technology, Faculty of Medical Technology, Mahidol University, Bangkok 10700, Thailand*

[3] *Department of Pharmaceutical Biosciences, Uppsala University, Uppsala SE751 24, Sweden*

*Corresponding author. E-mail: chanin.nan@mahidol.ac.th

# 1 Details on multivariate analysis

## 1.1 Principal component analysis (PCA)

PCA results in mutually orthogonal axes, called principal components (PCs), which are linearly uncorrelated. Two important features of PCA are the loadings and scores. The loadings reveal correlations between all variables simultaneously, whereas the scores reveal similarities and differences between samples. The fundamental assumption is that PCs with a high explained variance possess systematic variance, whereas PCs with a low explained variance represent noise. Thus, it is important to decide on the number of PCs that sufficiently represent the information present in the data. Including higher-order PCs may just over-fit a model and result in a poor generalization of the data structures.

## 1.2 Partial least squares (PLS)

PLS is a robust regression method that can handle a large amount of predictors without severely affecting the predictive power of its models. Briefly, PLS finds linear combinations of the predictors, called components or latent variables. The latent variables are chosen to maximally summarize the covariance with the response, thus yielding components that maximally summarize the variation of the data set in terms of the descriptors while simultaneously having these components correlated with the response. Therefore, PLS finds a compromise between predictor space dimension reduction and the predictability of the relationship with the response (i.e., $pIC_{50}$). Because PLS identifies the optimal predictor sample dimension reduction to perform regression with the response, it is important to select the optimal principle component. Each extracted component increases the explained variation of the predictors, where the first component normally identifies the real correlation between the predictors and response.

## 1.3 Random forest (RF)

The route towards an activity class begins at the root node, where it is then passed through decision nodes that require choices to be made based on the features (i.e., compound, protein and cross–terms). These outcomes split the data across branches that indicate the potential class of a decision. The final decision can be made when the tree terminated by leaf nodes provides a particular expected class as the result of a series of decisions. This provides tremendous insights into how the model works for a particular task of prediction, which makes it especially appropriate for classification. In RF, the classification is obtained by averaging the results of all tress by a majority vote based on each tree.

**Supplementary Table 1**

Summary of mutational information and $pIC_{50}$ of aromatase variants

| No. | Compound | Position on aromatase sequence | | | | | | | | | | | | | $pIC_{50}$ |
|---|---|---|---|---|---|---|---|---|---|---|---|---|---|---|---|
| | | 119 | 124 | 130 | 133 | 235 | 308 | 309 | 310 | 320 | 395 | 474 | 475 | 476 | |
| 1 | 4-OHA | K | C | K | I | F | P | A | T | F | I | I | H | D | 6.8729 |
| 2 | 4-OHA | K | C | K | I | L | P | D | T | F | I | I | H | D | 7.2441 |
| 3 | 4-OHA | K | C | K | W | F | P | D | T | F | I | I | H | D | 7.2757 |
| 4 | 4-OHA | K | C | K | Y | F | P | D | T | F | I | I | H | D | 7.1739 |
| 5 | 4-OHA | K | C | K | I | F | P | D | T | F | F | I | H | D | 7.5686 |
| 6 | 4-OHA | K | C | K | I | F | P | D | T | F | I | M | H | D | 6.9830 |
| 7 | 4-OHA | K | C | K | I | F | P | D | T | F | I | N | H | D | 6.9547 |
| 8 | 4-OHA | K | C | K | I | F | P | D | T | F | I | W | H | D | 7.1079 |
| 9 | 4-OHA | K | C | K | I | F | P | D | T | F | I | Y | H | D | 6.8013 |
| 10 | 4-OHA | K | C | K | I | F | F | D | T | F | I | I | H | D | 8.0000 |
| 11 | 4-OHA | K | C | K | I | F | P | D | S | F | I | I | H | D | 6.6990 |
| 12 | 4-OHA | K | C | K | I | F | P | D | T | F | I | I | H | D | 7.2218 |
| 13 | MDL101003 | K | C | K | I | F | P | A | T | F | I | I | H | D | 7.4559 |
| 14 | MDL101003 | K | C | K | I | L | P | D | T | F | I | I | H | D | 7.7447 |
| 15 | MDL101003 | K | C | K | W | F | P | D | T | F | I | I | H | D | 7.7959 |
| 16 | MDL101003 | K | C | K | Y | F | P | D | T | F | I | I | H | D | 7.6576 |
| 17 | MDL101003 | K | C | K | I | F | P | D | T | F | F | I | H | D | 7.8861 |
| 18 | MDL101003 | K | C | K | I | F | P | D | T | F | I | M | H | D | 7.7959 |
| 19 | MDL101003 | K | C | K | I | F | P | D | T | F | I | N | H | D | 7.7959 |
| 20 | MDL101003 | K | C | K | I | F | P | D | T | F | I | W | H | D | 7.9586 |
| 21 | MDL101003 | K | C | K | I | F | P | D | T | F | I | Y | H | D | 7.7959 |
| 22 | MDL101003 | K | C | K | I | F | F | D | T | F | I | I | H | D | 7.3372 |
| 23 | MDL101003 | K | C | K | I | F | P | D | S | F | I | I | H | D | 7.6990 |
| 24 | MDL101003 | K | C | K | I | F | P | D | T | F | I | I | H | D | 7.9208 |
| 25 | 7$\alpha$-APTADD | K | C | K | I | F | P | A | T | F | I | I | H | D | 6.5575 |
| 26 | 7$\alpha$-APTADD | K | C | K | I | L | P | D | T | F | I | I | H | D | 6.6402 |
| 27 | 7$\alpha$-APTADD | K | C | K | W | F | P | D | T | F | I | I | H | D | 6.5686 |
| 28 | 7$\alpha$-APTADD | K | C | K | Y | F | P | D | T | F | I | I | H | D | 6.6968 |
| 29 | 7$\alpha$-APTADD | K | C | K | I | F | P | D | T | F | F | I | H | D | 6.5901 |
| 30 | 7$\alpha$-APTADD | K | C | K | I | F | P | D | T | F | I | M | H | D | 6.8386 |
| 31 | 7$\alpha$-APTADD | K | C | K | I | F | P | D | T | F | I | N | H | D | 6.8041 |
| 32 | 7$\alpha$-APTADD | K | C | K | I | F | P | D | T | F | I | W | H | D | 6.9431 |
| 33 | 7$\alpha$-APTADD | K | C | K | I | F | P | D | T | F | I | Y | H | D | 6.9914 |
| 34 | 7$\alpha$-APTADD | K | C | K | I | F | F | D | T | F | I | I | H | D | 7.2218 |
| 35 | 7$\alpha$-APTADD | K | C | K | I | F | P | D | S | F | I | I | H | D | 6.6757 |
| 36 | 7$\alpha$-APTADD | K | C | K | I | F | P | D | T | F | I | I | H | D | 6.7721 |

**Supplementary Table 1**

Continued . . .

| No. | Compound | Position on aromatase sequence | | | | | | | | | | | | | pIC$_{50}$ |
|---|---|---|---|---|---|---|---|---|---|---|---|---|---|---|---|
| | | 119 | 124 | 130 | 133 | 235 | 308 | 309 | 310 | 320 | 395 | 474 | 475 | 476 | |
| 37 | Aminoglutethimide | K | C | K | I | F | P | A | T | F | I | I | H | D | 6.1367 |
| 38 | Aminoglutethimide | K | C | K | I | L | P | D | T | F | I | I | H | D | 5.7696 |
| 39 | Aminoglutethimide | K | C | K | W | F | P | D | T | F | I | I | H | D | 5.3188 |
| 40 | Aminoglutethimide | K | C | K | Y | F | P | D | T | F | I | I | H | D | 5.1308 |
| 41 | Aminoglutethimide | K | C | K | I | F | P | D | T | F | F | I | H | D | 5.4949 |
| 42 | Aminoglutethimide | K | C | K | I | F | P | D | T | F | I | M | H | D | 5.7696 |
| 43 | Aminoglutethimide | K | C | K | I | F | P | D | T | F | I | N | H | D | 5.6021 |
| 44 | Aminoglutethimide | K | C | K | I | F | P | D | T | F | I | W | H | D | 6.1367 |
| 45 | Aminoglutethimide | K | C | K | I | F | P | D | T | F | I | Y | H | D | 6.0655 |
| 46 | Aminoglutethimide | K | C | K | I | F | F | D | T | F | I | I | H | D | 5.2757 |
| 47 | Aminoglutethimide | K | C | K | I | F | P | D | S | F | I | I | H | D | 6.1739 |
| 48 | Aminoglutethimide | K | C | K | I | F | P | D | T | F | I | I | H | D | 5.2596 |
| 49 | CGS20267 | K | C | K | I | F | P | A | T | F | I | I | H | D | 8.0969 |
| 50 | CGS20267 | K | C | K | I | L | P | D | T | F | I | I | H | D | 8.8861 |
| 51 | CGS20267 | K | C | K | W | F | P | D | T | F | I | I | H | D | 8.7447 |
| 52 | CGS20267 | K | C | K | Y | F | P | D | T | F | I | I | H | D | 8.7212 |
| 53 | CGS20267 | K | C | K | I | F | P | D | T | F | F | I | H | D | 8.8239 |
| 54 | CGS20267 | K | C | K | I | F | P | D | T | F | I | M | H | D | 9.3979 |
| 55 | CGS20267 | K | C | K | I | F | P | D | T | F | I | N | H | D | 9.0458 |
| 56 | CGS20267 | K | C | K | I | F | P | D | T | F | I | W | H | D | 9.3979 |
| 57 | CGS20267 | K | C | K | I | F | P | D | T | F | I | Y | H | D | 9.3979 |
| 58 | CGS20267 | K | C | K | I | F | F | D | T | F | I | I | H | D | 8.7959 |
| 59 | CGS20267 | K | C | K | I | F | P | D | S | F | I | I | H | D | 9.0458 |
| 60 | CGS20267 | K | C | K | I | F | P | D | T | F | I | I | H | D | 8.8539 |
| 61 | Vorozole | K | C | K | I | F | P | A | T | F | I | I | H | D | 8.3188 |
| 62 | Vorozole | K | C | K | Y | F | P | D | T | F | I | I | H | D | 9.0969 |
| 63 | Vorozole | K | C | K | I | F | P | D | T | F | F | I | H | D | 9.3010 |
| 64 | Vorozole | K | C | K | I | F | P | D | T | F | I | Y | H | D | 9.6990 |
| 65 | Vorozole | K | C | K | I | F | F | D | T | F | I | I | H | D | 9.0000 |
| 66 | Vorozole | K | C | K | I | F | P | D | S | F | I | I | H | D | 9.3010 |
| 67 | Vorozole | K | C | K | I | F | P | D | T | F | I | I | H | D | 9.0458 |
| 68 | Anastrozole | K | C | K | I | F | P | A | T | F | I | I | H | D | 6.6990 |
| 69 | Anastrozole | K | C | K | I | L | P | D | T | F | I | I | H | D | 7.7447 |
| 70 | Anastrozole | K | C | K | W | F | P | D | T | F | I | I | H | D | 7.8239 |
| 71 | Anastrozole | K | C | K | Y | F | P | D | T | F | I | I | H | D | 7.2757 |
| 72 | Anastrozole | K | C | K | I | F | P | D | T | F | F | I | H | D | 7.4318 |

**Supplementary Table 1**

Continued . . .

| No. | Compound | Position on aromatase sequence | | | | | | | | | | | | | $pIC_{50}$ |
|---|---|---|---|---|---|---|---|---|---|---|---|---|---|---|---|
| | | 119 | 124 | 130 | 133 | 235 | 308 | 309 | 310 | 320 | 395 | 474 | 475 | 476 | |
| 73 | Anastrozole | K | C | K | I | F | P | D | T | F | I | M | H | D | 8.0969 |
| 74 | Anastrozole | K | C | K | I | F | P | D | T | F | I | N | H | D | 7.6990 |
| 75 | Anastrozole | K | C | K | I | F | P | D | T | F | I | W | H | D | 8.2218 |
| 76 | Anastrozole | K | C | K | I | F | P | D | T | F | I | Y | H | D | 8.0969 |
| 77 | Anastrozole | K | C | K | I | F | F | D | T | F | I | I | H | D | 7.4318 |
| 78 | Anastrozole | K | C | K | I | F | P | D | S | F | I | I | H | D | 8.0969 |
| 79 | Anastrozole | K | C | K | I | F | P | D | T | F | I | I | H | D | 7.5686 |
| 80 | MR20814 | K | Y | K | I | F | P | D | T | F | I | I | H | D | 5.3565 |
| 81 | MR20814 | K | C | K | I | F | P | D | T | F | I | I | H | E | 5.3298 |
| 82 | MR20814 | K | C | K | I | F | P | D | T | F | I | I | H | N | 5.6946 |
| 83 | MR20814 | K | C | K | I | F | P | D | T | C | I | I | H | D | 5.0000 |
| 84 | MR20814 | K | C | K | I | F | P | D | T | F | I | I | A | D | 6.0362 |
| 85 | MR20814 | K | C | K | I | F | P | D | T | F | I | T | H | D | 5.0000 |
| 86 | MR20814 | E | C | K | I | F | P | D | T | F | I | I | H | D | 5.0088 |
| 87 | MR20814 | V | C | K | I | F | P | D | T | F | I | I | H | D | 5.0000 |
| 88 | MR20814 | Y | C | K | I | F | P | D | T | F | I | I | H | D | 5.4248 |
| 89 | MR20814 | K | C | N | I | F | P | D | T | F | I | I | H | D | 6.6383 |
| 90 | MR20814 | K | C | K | I | F | P | D | T | F | I | I | H | D | 4.9654 |
| 91 | MR20492 | K | Y | K | I | F | P | D | T | F | I | I | H | D | 6.4815 |
| 92 | MR20492 | K | C | K | I | F | P | D | T | F | I | I | H | E | 5.2518 |
| 93 | MR20492 | K | C | K | I | F | P | D | T | F | I | I | H | N | 6.0362 |
| 94 | MR20492 | K | C | K | I | F | P | D | T | C | I | I | H | D | 5.2716 |
| 95 | MR20492 | K | C | K | I | F | P | D | T | F | I | I | A | D | 6.3468 |
| 96 | MR20492 | K | C | K | I | F | P | D | T | F | I | T | H | D | 5.0000 |
| 97 | MR20492 | E | C | K | I | F | P | D | T | F | I | I | H | D | 6.1308 |
| 98 | MR20492 | V | C | K | I | F | P | D | T | F | I | I | H | D | 5.2069 |
| 99 | MR20492 | Y | C | K | I | F | P | D | T | F | I | I | H | D | 5.2426 |
| 100 | MR20492 | K | C | N | I | F | P | D | T | F | I | I | H | D | 5.3696 |
| 101 | MR20492 | K | C | K | I | F | P | D | T | F | I | I | H | D | 5.4056 |
| 102 | MR20494 | K | Y | K | I | F | P | D | T | F | I | I | H | D | 6.4815 |
| 103 | MR20494 | K | C | K | I | F | P | D | T | F | I | I | H | E | 6.8239 |
| 104 | MR20494 | K | C | K | I | F | P | D | T | F | I | I | H | N | 6.8239 |
| 105 | MR20494 | K | C | K | I | F | P | D | T | C | I | I | H | D | 6.0269 |
| 106 | MR20494 | K | C | K | I | F | P | D | T | F | I | I | A | D | 7.0000 |
| 107 | MR20494 | K | C | K | I | F | P | D | T | F | I | T | H | D | 5.5528 |
| 108 | MR20494 | E | C | K | I | F | P | D | T | F | I | I | H | D | 6.2757 |

**Supplementary Table 1**

Continued . . .

| No. | Compound | Position on aromatase sequence | | | | | | | | | | | | | $pIC_{50}$ |
|---|---|---|---|---|---|---|---|---|---|---|---|---|---|---|---|
| | | **119** | **124** | **130** | **133** | **235** | **308** | **309** | **310** | **320** | **395** | **474** | **475** | **476** | |
| 109 | MR20494 | V | C | K | I | F | P | D | T | F | I | I | H | D | 5.8097 |
| 110 | MR20494 | Y | C | K | I | F | P | D | T | F | I | I | H | D | 6.7696 |
| 111 | MR20494 | K | C | N | I | F | P | D | T | F | I | I | H | D | 6.6990 |
| 112 | MR20494 | K | C | K | I | F | P | D | T | F | I | I | H | D | 6.6383 |

**Supplementary Table 2**

List of SMILES notation for aromatase inhibitors used herein

| | Compound | SMILES |
|---|---|---|
| 1 | 4-OHA | C[C@]12CCC3C(CCC4=C(O)C(=O)CC[C@]34C)C1CCC2=O |
| 2 | MDL101003 | C[C@]12CCC3C(CCC4=CC(=O)[C@H]5COC[C@@]34C5)C1CCC2=O |
| 3 | 7$\alpha$-APTADD | C[C@]12CCC3C(C1CCC2=O)C(CC1=CC(=O)C=C[C@]31C)SC1=CC=C(N)C=C1 |
| 4 | Aminoglutethimide | CCC1(CCC(=O)NC1=O)C1CCC(N)CC1 |
| 5 | CGS20267 | [C-]#[N+]C1=CC=C(C=C1)C(N1C=NC=N1)C1=CC=C(C=C1)[N+]#[C-] |
| 6 | Vorozole | CN1N=NC2=C1C=C(C=C2)C(N1C=NC=N1)C1=CC=C(Cl)C=C1 |
| 7 | Anastrozole | CC(C)([N+]#[C-])C1=CC(=CC(CN2C=NC=N2)=C1)C(C)(C)[N+]#[C-] |
| 8 | MR20814 | [H]C([H])(O)C1=CC2=C(C=C1)C(=O)C(CC1=CC=NC=C1)=C2N |
| 9 | MR20492 | ClC1=CC=C(C=C1)C1CN2C=CC=C2C(=O)\C1=C/C1=CC=NC=C1 |
| 10 | MR20494 | ClC1=CC=C(C=C1)C1CN2C=CC=C2C(=O)\C1=C/C1=CN=CC=C1 |
